# Are we along the path for laparoscopic partial nephrectomy?

**Published:** 2007

**Authors:** Naval Khurana, Saurabh Agarwal, Aneesh Srivastava

**Affiliations:** Department of Urology and Renal Transplant, SGPGIMS, Lucknow, India. E-mail: drrgoyal@yahoo.com

## SUMMARY

This is the first study addressing five-year outcomes of laparoscopic partial nephrectomy (LPN) in 56 patients, each of whom has completed a minimum of five years of follow-up. Ninety-one per cent of patients had incidentally discovered renal masses with a mean tumor size of 2.9 cm, which were benign in 34% and malignant in 66% and pathological tumor stage was pT1a in 32 (86%). Final surgical margin was positive for cancer in one patient. Median serum creatinine preoperatively and postoperatively was 0.9 and 1.0 mg/dl, respectively. No patient with normal baseline serum creatinine undergoing elective laparoscopic partial nephrectomy had postoperative chronic renal insufficiency (serum creatinine more than 2 mg/dl). At a median follow-up of 5.7 years (range 5.0 to 6.9) no distant recurrence (0%) and a single local recurrence (2.7%) were detected. Overall and cancer-specific survival was 86% and 100%, respectively, at five years.

## COMMENTS

Laparoscopic partial nephrectomy is technically feasible with exceptional results at certain high-volume centers of excellence. Nephron spring surgery (NSS) is still greatly underused worldwide despite superior renal functional outcomes[1] and equivalent oncological outcomes compared to radical nephrectomy.[1,3] This is the greatest challenge facing NSS of any type. Factors preventing worldwide spread are bleeding from vessels and complications associated with opening of pyelocalyceal system. Various hemostatic techniques including hemostatic agents[4] (BioGlue and fibrin glue) have been used to overcome these problems with varying results. None of these pharmaceutical hemostatic agents have been reliably successful in sealing vessels or the collecting system, clinically. To be sure, the “tipping point” for spreading laparoscopic partial nephrectomy throughout the world would occur if the procedure could be reduced to a few simple steps, namely clamp the renal vessels, cut out the tumor, dry the exposed renal surface, layer on the hemostatic agent, wait few minutes, unclamp and close. While we are not there yet, surely we are well along the path.
